# Comparing Family Health Before and After a Family-Focused Nutrition Program during the Pandemic

**DOI:** 10.1007/s10995-024-03934-2

**Published:** 2024-06-19

**Authors:** Margaret Rose Mahoney, Evan C Sommer, Filoteia Popescu, Laura E Adams, Shari Barkin

**Affiliations:** 1 Vanderbilt University School of Medicine, 1161 21st Ave S # D3300, Nashville, TN 37232 USA; 2 Vanderbilt University Medical Center, 1211 Medical Center Dr, Nashville, TN 37232 USA; 3 The University of Tennessee Health Science Center College of Medicine, 910 Madison Ave #1002, Memphis, TN 38103 USA; 4 Children’s Hospital of Richmond at VCU, 1000 E Broad St, Richmond, VA 23219 USA

**Keywords:** Family health, Nutrition, Health equity

## Abstract

**Introduction:**

The COVID-19 pandemic affected child health behaviors, leading to worse physical health. Given the importance of good family health in improved child health outcomes, this secondary cohort analysis tested the hypothesis that family health would improve from baseline to 12-week follow-up after participation in a novel family nutrition program.

**Methods:**

Diverse parent-child dyads participated in a home-based virtual Teaching Kitchen Outreach (vTKO) program (11 weekly healthy, low-cost recipes, cooking videos, and associated groceries delivered). The primary outcome was the Family Healthy Lifestyle Subscale (FHLS). Secondary outcomes were parent and child nutrition, and food insecurity. Statistical testing and modeling were used to evaluate pre-post outcomes.

**Results:**

Of 123 enrolled dyads, 114 (93%) had sufficient data for analysis. Participants were 11% Hispanic, 54% Black, and 28% White; 31% completed high school or less; and 30% indicated food insecurity. Cohort mean pre-post FHLS scores significantly increased (25.5 vs. 27.3; *p* < 0.001). There were significant improvements in parent nutrition (*p* < 0.001) and child nutrition (*p* = 0.02 to < 0.001), but not in food security. After adjusting for baseline covariates, tobit regression found statistically significant pre-post FHLS differences (2.3; 95% CI=[1.4, 3.3]; *p* < 0.001).

**Discussion:**

Participants in the novel home-based vTKO program reported improved family health over 12 weeks.

## Significance

While nutrition programs can improve individuals’ behavior, it is not known how they impact the health of the family unit. This home-based virtual nutrition program provided groceries and taught food management skills to underserved families, examining family health differences over time. Family health is a proximal social determinant of health that predicts risk or resilience during times of stress. This is the first study to examine a change in family health over time.

## Introduction

The Corona Virus 2019 (COVID-19) national emergency was a stressor to many, leading people to depend on their families even more than before. The pandemic impacted health behaviors, leading to worse child nutrition and eating patterns, particularly in vulnerable groups (Gauvin et al., 2022; Teixeira et al., 2021; Philippe et al. 2021; Xiang et al., 2020). Pandemic-associated stress affected parenting and was associated with higher use of non-nutritious snacks and irregular snacking times (Jansen et al., 2021). Food insecurity in the United States increased during the pandemic, and families with children were at an increased risk of becoming newly food insecure (Niles, 2020; Janda et al., 2021) In some states, such as Tennessee, the rate of food insecurity increased nearly four-fold for families with children (Patrick et al. 2020). Food insecurity is associated with obesity, which leads to a host of health comorbidities across the lifespan (Dhurandhar, 2016). Janda et al. stated that “the COVID-19 pandemic did not create new food insecurity disparities. Rather, the pandemic exacerbated pre-existing disparities” (2021). Additionally, in-person healthcare services and community-based programming were challenging to deliver due to public health guidelines (Dubey et al., 2020). Taken together, the pandemic negatively impacted family health, especially under-resourced families who were already vulnerable to poor nutrition and food insecurity.

Family health is a strong predictor of risk or resilience, and some consider it to be a proximal social determinant of health, contributing to child health outcomes (Chu et al., 2021; Prime et al., 2020; Barnes et al., 2020; Crandall et al., 2020). Prior research intervenes at the level of the family and demonstrates improved child nutrition with increased vegetables and healthy food availability (Berge et al., 2021). However there are very few interventions aimed at improving the health of the family. Interventions that strengthen family health have the potential to buffer against life’s stresses. While family health is recognized as a critical component for either exacerbating or augmenting children’s health, there are few tools to measure family health and limited studies report family health, as opposed to individual health, as a direct outcome (Ramaswami et al., 2022). Crandall et al. developed and validated the Family Health Scale (FHS) to fill this gap (2020). In this study, the Family Healthy Lifestyle Subscale is used for the first time to measure improvements in family health over time.

A randomized controlled trial (RCT) was conducted to understand the impact of a virtual adaptation of a health coaching program for families with young children (Popescu et al., 2022) All parent-child dyads received a home-based virtual Teaching Kitchen Outreach (vTKO) program, including weekly recipes and associated groceries delivered to their home, and half were randomly assigned to also receive virtual health coaching. The RCT found that the virtual health coaching did not lead to a significant improvement in family health or resilience compared to the vTKO program. Additional details about the RCT and primary analysis of the intervention effect are presented elsewhere (Popescu et al., 2022) This is a secondary cohort analysis to explore whether participation in the home-based vTKO program was associated with significant improvements on measures of family health, parent and child nutrition, and food security across all participating dyads from baseline to 12-week follow-up, regardless of random assignment.

## Methods

The study (NCT05328193) was approved by the Vanderbilt Institutional Review Board (IRB #200,257) in accordance with the Declaration of Helsinki and funded by the Joe C. Davis Foundation. Written informed consent was completed by all participants prior to study inclusion.

### Recruitment

Parent-child dyads were recruited on a rolling basis from the Vanderbilt Pediatric Primary Care Clinic (VPPCC) and community organizations that serve under-resourced families in Nashville, Tennessee. Families initiated the 12-week study between March and August 2021 and completed follow-up data collection between June and November 2021. Recruitment methods included flyers, emails, phone calls, and texts. Eligible families participated in programming with one of the participating organizations, had a parent age ≥ 18 years, a child aged 2–8 years, and the ability to participate in virtual programming.

### Study Description

This study utilized components from previously tested behavioral interventions, which were adjusted to the context of the COVID-19 pandemic (Barkin et al., 2018; Heerman et al., 2020, 2021; Smith et al., 2020). All families received an adapted home-based virtual Teaching Kitchen Outreach (vTKO) program over 12 weeks, in which they first received program instructions and starter cooking utensils for parent-child pairs to use in cooking (e.g. cutting board, measuring spoons) followed by 11 subsequent weekly recipes, cooking videos, and associated groceries (Heerman et al., 2020). The program was designed to engage both the parent and child together in preparing healthy meals. The recipes were previously tested with children to ensure palatability, and the associated groceries consisted of ingredients that can be purchased with the Supplemental Nutrition Assistance Program (SNAP) or The Special Supplemental Nutrition Program for Women, Infants, and Children (WIC) (Food and Nutrition Service, 2018; Oliveira & Frazao, 2015). Half of families were randomized to additionally receive weekly virtual health coaching, but this secondary cohort analysis examines pre-post differences across all participants, regardless of assignment. Participants completed surveys at baseline and at 12-week follow-up, after the program ended. All surveys were parent-reported and completed online or over the phone with a certified data collector.

### Measurements and Outcomes

Demographics: Demographics about the child, parent, and family unit were collected at baseline. Child age was reported in years. Parent education was categorized binarily (high school or less; more than high school); parent race was categorized as White, Black, Hispanic, or Multiple races or ethnicities; number of adults in the home was categorized binarily (one; more than one).

vTKO Program Participation: Process measures included the number of successful grocery deliveries and the number of participant-reported vTKO recipes prepared (both range 0–11).

**Fig. 1:**
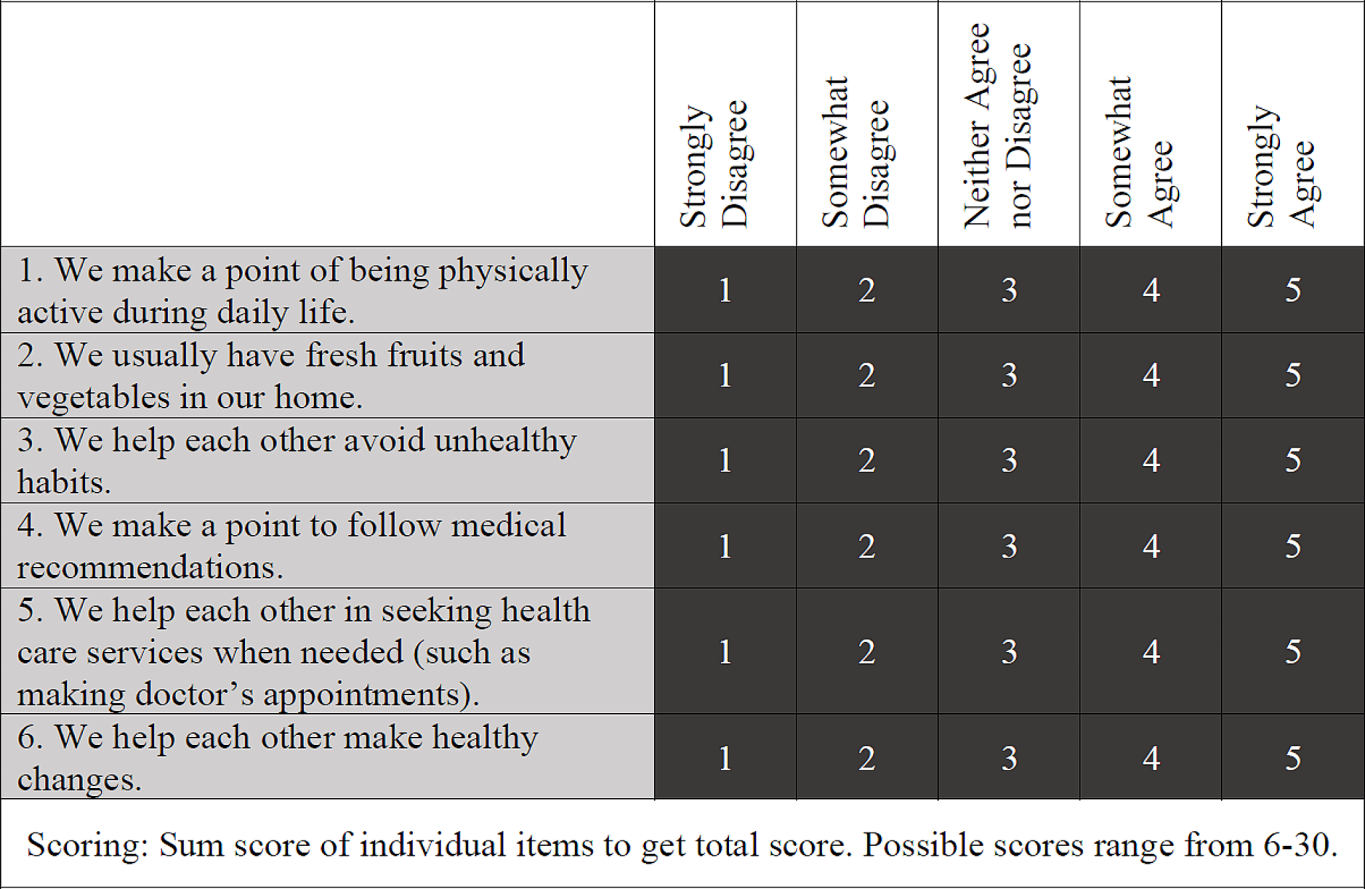
Family Healthy Lifestyle Subscale (FHLS) survey

Primary Outcome: The primary outcome of family health was measured by the Family Healthy Lifestyle Subscale (FHLS) of the FHS (Crandall et al., 2020). The FHLS total score ranges from 6 to 30, with higher scores representing better family health, and it is calculated by summing across six 5-point Likert scale items (strongly disagree to strongly agree) [Fig. 1].

Of the four validated FHS domains, the FHLS domain was selected as the primary outcome because it aligned with the healthy behaviors encouraged by the program (e.g., diet and health behavior decision-making).

Secondary Outcomes: Secondary outcomes included measures of parent and child nutrition and food security at baseline and 12-week follow-up. Parent nutrition was measured using the Starting the Conversation: Dietary Scale (Paxton et al., 2011). It is a validated, 8-item instrument to assess diet, with lower scores indicating better nutrition (score range 0–16). Child nutrition was assessed based on their 30-day consumption of six food categories from the Centers for Disease Control Dietary Screener, which contains items used widely on the Behavioral Risk Factor Surveillance System (Marks, 1985). Frequency of consumption within the following six food categories was analyzed: sugary beverages (combined soda and sweetened drinks), snacks, sweets, fruits, leafy greens, and other vegetables. Food security was measured using the United States Department of Agriculture Household Food Security Scale, which is a validated, 6-item instrument that categorizes households as food secure or food insecure (Blumberg et al., 1999).

### Statistical Methods

Participant characteristics and outcomes are presented as mean (SD), median (Q1, Q3), or frequency (%) for categorical variables. The primary outcome (FHLS) was initially analyzed for simple pre-post mean differences using two-sample paired *t*-tests. A multilevel mixed-effects tobit regression model with an upper limit of 30 was also used to determine whether baseline FHLS scores were significantly different from follow-up scores after adjusting for baseline covariates in the model.^1^ Tobit regression (also called censored regression) with an upper limit was more appropriate than an ordinary least squares approach for analyzing the FHLS outcome because of the substantial degree of left skew observed in the data, indicating a possible ceiling effect. The multilevel framework utilized two levels (time nested within individual participant) to account for there being up to two measurements of the outcome per participant. Baseline was determined to be different from follow-up by examination of the time coefficient’s significance. It is important to note that tobit model raw coefficients are interpreted as though there was no ceiling or upper limit on the outcome, which is often unrealistic (e.g., they can produce estimates outside the possible outcome range). Therefore, while the results are presented as raw coefficients with 95% confidence intervals (CIs) and *p*-values for reference, a model-based estimate of the baseline-follow-up FHLS difference with an upper limit of 30 imposed is also presented to facilitate interpretation. Covariates in the model included an indicator for time (baseline; 12-week follow-up), randomized group (vTKO only; vTKO plus virtual coaching), child baseline age (years), parent education (high school or less; more than high school), food security (secure; insecure), parent race (White, Black, Hispanic, or Multiple races or ethnicities), and number of adults in the home (one; more than one). To determine whether there were significant pre-post differences among the six FHLS items, a post-hoc analysis evaluated for mean differences on each item using two-sample paired *t*-tests. The parent nutrition secondary outcome was also analyzed with this approach. However, due to the effects of numerous consumption frequency responses of zero on the distributions, a common occurrence in frequency variables, child nutrition was evaluated using nonparametric Wilcoxon matched-pairs signed-rank tests. Binary food security status at baseline and follow-up was evaluated using McNemar’s chi-squared test for the difference in proportions between paired data. Secondary analyses were conducted on the subset of participants with full data at baseline and follow-up. Statistical analyses were conducted using Stata version 17.0 (StataCorp). Statistical significance was defined as a two-sided p-value less than 0.05, and marginal statistical significance was defined as a two-sided p-values less than 0.1.

## Results

### Recruitment

Of the 123 parent-child dyads randomized, 114 (93%) had sufficient data for inclusion in the primary multilevel mixed-effect tobit regression analysis with 58 (51%) assigned to the vTKO only group and 56 (49%) assigned to the vTKO plus virtual coaching group. One hundred and nine families (89%) had sufficient data for inclusion in the pre-post analyses.

### Participant Baseline Characteristics

Participant characteristics for the cohort are described in [Table 1].

**Table 1:** Participant baseline characteristics

	*N* = 114
Randomized group	
vTKO only	58 (50.9%)
vTKO plus virtual coaching	56 (49.1%)
Mean child age (years)	5.7 (1.7)
Parent education	
High school or less	35 (30.7%)
More than high school	79 (69.3%)
Household food security	
Food secure	80 (70.2%)
Food insecure with or without hunger	34 (29.8%)
Parent race or ethnicity	
White	32 (28.1%)
Black	62 (54.4%)
Hispanic	13 (11.4%)
Multiple races or ethnicities	7 (6.1%)
Household number of adults	
One adult	45 (39.5%)
More than one adult	69 (60.5%)
Family Healthy Lifestyle Subscale (range: 6–30)	25.5 (4.0)

Overall, children had a mean age of 5.7 (1.7) years. Parents were 28% White, 54% Black, 11% Hispanic, and 6% were of multiple races or ethnicities, 31% had a high school education or less, 40% reported single parent homes, and 30% indicated food insecurity.

### Intervention Participation

Among the 123 enrolled families, 117 (95%) received all 11 grocery deliveries. Reasons for not receiving a delivery included allergies to a key ingredient or changed address. With respect to recipe preparation, among 108 reporting families, 92 (85%) reported making at least 6 out of 11 recipes and 42 (39%) reported making all recipes.

### Analyses of Primary Outcome

**Fig. 2:**
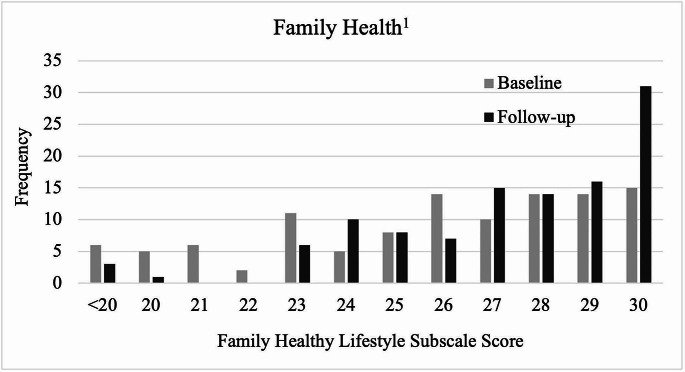
Distribution of family healthy lifestyle subscale scores at baseline and 12-week follow-up. ^1^ Items were on a 5-point Likert scale from strongly disagree to strongly agree. Analytic sample size for this figure was *n* = 110

The baseline FHLS distribution was skewed left with a mean of 25.5 (4.5), on a scale of 6–30 [Fig. 2].

Mean FHLS score statistically significantly improved from baseline to follow-up (25.5 vs. 27.3, respectively; *p* < 0.001). For reference, the raw coefficient for time in the multilevel tobit regression model was statistically significant (2.3; 95% CI=[1.4, 3.3]; *p* < 0.001), indicating that follow-up FHLS improved from baseline after adjusting for covariates [Table 2].

**Table 2:** Multilevel mixed-effects tobit regression model^a^

	Coefficient	95% confidence interval	*p*-value
Time (baseline vs. follow-up)	2.3	[1.4, 3.3]	< 0.001
Randomized group (vTKO only vs. vTKO plus virtual coach)	0.6	[-0.6, 1.9]	0.3
Baseline child age (years)	0.2	[-0.2, 0.6]	0.3
Parent education (high school or less vs. more than high school)	-1.6	[-3.0, -0.2]	0.03
Food security (food secure vs. food insecure)	-0.3	[-1.7, 1.0]	0.6
Parent race/ethnicity (ref: White)			
Black	-1.4	[-2.9, 0.2]	0.09
Hispanic	-1.0	[-3.2, 1.3]	0.4
Multiple races or ethnicities	0.9	[-1.9, 3.7]	0.5
Number of adults in home (one vs. more than one)	-1.3	[-2.7, 0.2]	0.08

^a^ The multilevel mixed-effects tobit regression model had two levels (time nested within individual participant) to account for repeated outcome measures. The upper limit was specified as 30 (the maximum possible score on the FHLS). Baseline was determined to be significantly different from follow-up by a statistically significant coefficient for the time variable

However, this raw coefficient does not account for the ceiling or upper limit on the outcome. To facilitate a more realistic interpretation, the model-based estimate for this improvement with the upper limit of 30 imposed is also presented (1.8; 95% CI=[1.0, 2.5]; *p* < 0.001). Additionally, there was a significant association of higher parent education with lower FHLS score (-1.6; 95% CI=[-3.0, -0.2]; *p* = 0.03). The model was unable to detect significant associations between FHLS score and the other covariates. A post-hoc analysis of the six individual items that make up the FHLS found significant differences in all but one item, which was marginally statistically significant [Table 3].

**Table 3:** Family healthy lifestyle subscale individual item baseline and follow-up comparisons^a^

Item	Baseline	Follow-up	*p*-value
1. We make a point of being physically active during daily life.	4.1 (1.0)	4.4 (0.8)	0.002
2. We usually have fresh fruits and vegetables in our home.	4.5 (0.9)	4.7 (0.5)	0.004
3. We help each other avoid unhealthy habits.	3.6 (1.2)	4.2 (0.8)	< 0.001
4. We make a point to follow medical recommendations.	4.5 (0.8)	4.6 (0.6)	0.07
5. We help each other in seeking health care services when needed (such as making doctor’s appointments).	4.6 (1.0)	4.8 (0.5)	0.006
6. We help each other make healthy changes.	4.3 (0.9)	4.6 (0.6)	< 0.001

^a^ Items were on a 5-point Likert scale from strongly disagree to strongly agree. Analytic sample size for these analyses was *n* = 109. Values are reported as mean (SD), and the *p*-values are from two-sample paired t-tests of mean equality

### Analyses of Secondary Outcomes

Among the subset of participants with sufficient data for secondary analyses (*n* = 109), mean parent nutrition improved (score decreased) from baseline to follow-up (7.5 vs. 6.0; *p* < 0.001). In tests of distributional equality, the 30-day child consumption frequencies for sugary beverages, snacks, and sweets significantly improved (decreased) from baseline to follow-up [Table 4].

**Table 4:** Child nutrition baseline and follow-up comparisons

Food category^a^	Baseline	Follow-up	*p*-value
Sugary beverages	8 (1, 38)	4 (0, 20)	< 0.001
Snacks	16 (8, 30)	12 (5, 20)	< 0.001
Sweets	12 (4, 30)	8 (4, 16)	< 0.001
Fruits	30 (20, 90)	60 (24, 60)	0.8
Leafy greens	8 (0, 16)	12 (4, 30)	0.02
Other vegetables	12 (4, 30)	20 (8, 30)	0.02

^a^ Items were parent-reported 30-day frequencies of child consumption of each food category. Analytic sample size for these analyses was *n* = 109. Values are reported as median (Q1, Q3), and the *p*-values are from Wilcoxon matched-pairs signed-rank tests of distributional equality

The distributions for child consumption of leafy greens and other vegetables also significantly improved (increased) from baseline to follow-up, while improvement in fruit consumption was not detectable as statistically significant. Among those reporting (*n* = 102), the percentage of families experiencing food insecurity decreased in the cohort, from 28.4 to 23.5%, although the difference between these proportions was not statistically significant (*p* = 0.3).

## Discussion

By providing food to families’ doorsteps alongside food management skills like cooking, the home-based virtual Teaching Kitchen Outreach (vTKO) program was associated with improved family health and child and parent nutrition in the context of the COVID-19 pandemic. The intervention demonstrated high fidelity, with most families using the provided groceries for the vTKO recipes. Taken together, these results suggest that the home-based vTKO program may be effective in promoting healthy lifestyle changes for families with young children.

The family provides a critical context that can influence child health and development, and family health is thought to be a proximal social determinant of health with the potential to buffer against poor child health outcomes (Prime et al., 2020; Barnes et al., 2020; Crandall et al., 2020). The American Academy of Pediatrics recommends family-based child obesity interventions, and prior research demonstrates that family-centered interventions are successful in improving individuals’ health behaviors (Smith et al., 2017, 2020; Ash et al., 2017). Although previous studies report effective interventions that either focus on child or parent health outcomes, no study to the authors’ knowledge has examined a program to improve the health of the family unit. The current study advances the field by testing an innovative family-based approach that acknowledges the influence that parents and children have on each other’s health (Faught et al., 2016; Savage et al., 2007; Swindle et al., 2020).

Due to the cohort design of the study, the observed improvement in family health cannot be causally linked to participation in the vTKO program. However, this improvement may be due to the family-focused approach where both parent and child were encouraged to actively participate in vTKO recipe preparation together. This approach was deliberate, using kid-tested recipes and low-tech tools. Preparing the recipes together was intended to foster engagement, helping both parent and child follow healthy eating practices. By addressing the health of the family unit together, family-centered programs, such as this, have the potential to break multigenerational cycles of poor health. For this reason, future work should assess whether the home-based vTKO program leads to sustained improvement of family health over time.

A surprising finding of the regression model was the significant negative association of a higher parent education level with a lower FHLS score. Prior research demonstrates that parent education is a powerful influencer of child health and development (Protano et al., 2017; Chi et al., 2017). Future research should explore this finding with a larger, more educationally diverse, sample or with an instrument that has greater variability. Analyzing an intervention effect was not the purpose of this cohort analysis and is reported elsewhere (Popescu et al., 2022).

In addition to the significant increase in family health, further evidence supporting the potential benefits of participation in the program is provided by the observed improvements in parent and child nutrition. This also demonstrates that improved family health can occur alongside individual healthy behavior change. The field of advancing family health is relatively new as an outcome, and it will be important to better understand how the whole (i.e., the family) might be different than the sum of its parts (i.e., the individuals within the family) (Ramaswami et al., 2022).

Access to healthy food is necessary but not sufficient to improve nutrition and health outcomes (Cummins et al., 2014; Adedokun et al., 2021; Na et al., 2021). Food security worsened during the pandemic, and it is evident that food insecurity leads to poor health outcomes (Niles, 2020; Janda et al., 2021; Tester et al., 2020). However, prior research demonstrates that food management skills, like meal planning, food preparation, and cooking, have a large impact on nutrition and may mitigate the impacts of food insecurity on child diet and parent feeding practices (Adedokun et al., 2021; Na et al., 2021; Ryan-Ibarra et al., 2020). By providing weekly groceries along with a short teaching video on food preparation, the home-based vTKO program addressed *both* food security and food management. Although not statistically significant, food security descriptively improved in the cohort during a time when it worsened for many families. For this reason, future studies should continue to offer practical solutions to effectively improve diet quality and promote sustained healthy behavior changes, particularly for underserved families with children. Moreover, while many virtually delivered programs originated out of necessity during the COVID-19 pandemic, they are now a new expectation for many children and families. Going forward, studies should continue to explore how this program and others like it can be leveraged to increase equity and accessibility of family-centered nutrition.

An important historical note is that advance payments of the Child Tax Credit became available on July 15, 2021, which occurred about half-way through the time frame of this study. Early research demonstrates that the first advance tax credit may have reduced food insufficiency in households with children (Shafer et al., 2022). However, five of the six items that make up the primary outcome (FHLS) measure aspects of family health are *not* directly tied to family financial resources. These five items measure family physical activity, avoidance of unhealthy habits, adherence to medical recommendations, seeking health care services when needed, and supporting each other in making healthy changes. However, the sixth item, “We usually have fresh fruits and vegetables in our home.” could be affected by financial resources, as well as by family preference. A post-hoc analysis of the six individual FHLS items demonstrated statistically significant improvement for five items, including the item regarding healthy food in the home. The one marginally significant item, “we usually adhere to medical recommendations”, assessed a behavior that was not a goal of the program. Obtaining improvements across non-financially related items suggests that while advance payment of the Child Tax Credit may have contributed to the positive outcomes observed, it was likely not solely responsible for them.

## Limitations

Despite the ability of the multilevel mixed-effects model to adjust for potentially confounding covariates when assessing baseline to follow-up FHLS differences, it is important to acknowledge that the cohort design of this study does not permit causal conclusions about the program’s effects. However, the significant FHLS improvement over time was an encouraging finding that merits further research into this important construct. Future studies seeking to incorporate the FHLS would benefit from additional research analyzing its relationships to other validated and/or objectively measured outcomes. Characterization of such relationships would help contextualize findings like the 2.2-point increase observed in the current study. Additional limitations include a potential ceiling effect for high FHLS scores, limiting the ability for families to improve over time. The pandemic was a tumultuous time and many contextual factors may have changed for families over the 12 weeks of study participation that were not accounted for in this study design. Potential selection bias caused by parents who chose to enroll in the study may have skewed the sample to overrepresent highly motivated individuals. However, participants represented racial/ethnic diversity and household structure diversity. Finally, because measures were captured via survey, self-report and social desirability biases could occur.

## Conclusions

This novel home-based nutrition program was associated with improved measures of family and individual health. Family health is an understudied construct, that could provide a new way forward in amplifying health outcomes.

## Data Availability

Not applicable.

## Footnotes

1 Tobit regression coefficients are interpreted similarly to ordinary least squares regression coefficients (i.e., a one-unit increase in a covariate is associated with a change in the outcome equal in magnitude to the corresponding coefficient). It is important to note that coefficients are interpreted as though there were no upper limit. Model-based estimation can be used to obtain estimates that are informed by the upper limit (see results section).

## Code Availability

Software application.
